# A Systematic Review: Family Support Integrated with Diabetes Self-Management among Uncontrolled Type II Diabetes Mellitus Patients

**DOI:** 10.3390/bs7030062

**Published:** 2017-09-15

**Authors:** Rian Adi Pamungkas, Kanittha Chamroonsawasdi, Paranee Vatanasomboon

**Affiliations:** 1 Department of Family Health, Mahidol University, Bangkok 10400, Thailand; adirian491@yahoo.com; 2 Department of Nursing, College of Health, Mega Rezky Makassar, Makassar 90245, Indonesia; 3 Department of Health Education and Behavioral Science, Mahidol University, Bangkok 10400, Thailand; paranee.vat@mahidol.ac.th

**Keywords:** self-management, family support, uncontrolled glycaemia, type 2 DM, systematic review

## Abstract

The rate of type-2 diabetes mellitus (T2D) is dramatically increasing worldwide. Continuing diabetes mellitus (DM) care needs effective self-management education and support for both patients and family members. This study aimed to review and describe the impacts of diabetes mellitus self-management education (DSME) that involve family members on patient outcomes related to patient health behaviors and perceived self-efficacy on self-management such as medication adherence, blood glucose monitoring, diet and exercise changes, health outcomes including psychological well-being and self-efficacy, and physiological markers including body mass index, level of blood pressure, cholesterol level and glycemic control. Three databases, PubMed, CINAHL, and Scopus were reviewed for relevant articles. The search terms were “type 2 diabetes,” “self-management,” “diabetes self-management education (DSME),” “family support,” “social support,” and “uncontrolled glycaemia.” Joanna Briggs Institute (JBI) guidelines were used to determine which studies to include in the review. Details of the family support components of DSME intervention and the impacts of these interventions had on improving the health outcomes patients with uncontrolled glycaemia patients. A total of 22 intervention studies were identified. These studies involved different DSME strategies, different components of family support provided, and different health outcomes to be measured among T2D patients. Overall, family support had a positive impact on healthy diet, increased perceived support, higher self-efficacy, improved psychological well-being and better glycemic control. This systematic review found evidence that DSME with family support improved self-management behaviors and health outcomes among uncontrolled glycaemia T2D patients. The findings suggest DSME models that include family engagement can be a useful direction for improving diabetes care.

## 1. Introduction

Worldwide prevalence of type II diabetes mellitus (T2D) is steadily increasing. In 2014, the World Health Organization (WHO) (2014) estimated that 422 million people have been diagnosed with DM globally [1]. In the Asian region, the estimated percentage of T2D could be more than 60% of people by 2030 [2]. Only 14.3% of the total diabetes patients meet the target goals for good glycemic control while a staggering 85.7% fail to meet target goals of glycemic control as measured by Hemoglobin A1c (HbA1c) level [3].

The HbA1c level is a commonly used for glycemic control among T2D patients. It is an endocrine test averaging blood sugar concentrations over the previous three months [4]. HbA1c levels greater than 7% are considered uncontrolled glycaemia [5].

Uncontrolled glycaemia T2D patients are associated with serious multiple long-term complications, including constriction of blood vessels, nephropathy and retinopathy, peripheral neuropathy, and problems of the cardiovascular system [6]. Factors linked with uncontrolled glycaemia in T2D patients include unhealthy eating habits, physical inactivity, nonadherence to medication, and lack of regular blood glucose monitoring [7].

Managing DM is the cornerstone of preventing long-term complications and improving the quality of life among people with T2D [8]. The American Diabetes Association (ADA) has presented Diabetes self-management education (DSME) as a foundation for good diabetes care. One reason is that DSME is so important because managing T2D is complex. Patients are given multiple tasks: they have to attend medical appointments regularly, adhere to verifying medication regimens and engage in self-care behaviors including at-home blood glucose monitoring, healthy dietary changes, and increased physical activity [3]. However, it is often difficult for people to consistently engage in various health behaviors necessary for good glycemic control. Common barriers include competing for daily demands, frustration, other emotional distress, and low self-commitment [9]. In addition, lack of knowledge, low levels of self-efficacy to successfully complete an activity, and insufficient social support from family members has been associated with poor diabetes self-management [10]. Some studies reported that family support has a positive effect on patients’ self-management behaviors [10,11].

Because people with DM2 are nested within their families and larger social environment, these factors can also influence a patient’s diabetes care. Family members are key sources of both instrumental and emotional support. Instrument support includes helping patients’ complete specific tasks, such as making an appointment with health care providers or helping with insulin injections while, emotional support can include providing comfort and encouragement when patients face distress or frustration over the long course of their diabetes care [12,13]. Recognizing the influence family can have diabetes care guidelines include the provision of diabetes education to family members or incorporate family support as part of the patient’s diabetes care plan. In this way, educations programs that focus only on the individual could be limited.

Though the benefit of family support is commonly discussed, few comprehensive reviews have explicitly explored this issue in the DSME literature. Therefore, this study aims to review DSME interventions that emphasize family support as a fundamental concept to enhance patients’ self-management, describe their components, and examine the relationship between DSME with family support interventions and diabetes-related outcomes among patients with T2D.

## 2. Objective

The study aimed to review and describe the impact of DSME that involves family members on patient outcomes related to patient health behaviors such as medication adherence, blood glucose monitoring, diet and exercise changes, psychological well-being and self-efficacy, and physiological markers including body mass index, blood pressure, cholesterol level and glycemic control.

## 3. Methods

This review described the impact of family involvement in DSME among patients with uncontrolled glycaemia. We used the PRISMA (Preferred Reporting Items for Systematic Reviews and Meta-Analysis) statement of all stages of the review. Three searches were conducted, yielding 675 articles after duplication removed. For all initial strategies, family support, social support, and uncontrolled glycaemia were the main search terms and were entered as the medical subject heading (MeSH) in the abstract and title field. Titles were eliminated if the research involved type 1 diabetes or gestational diabetes, or were not written in English. This produced 102 abstracts to examine for full article review. This initial review includes 23 articles that almost have relevance to the systematic review.

### 3.1. Eligibility Criteria

The PICO (Participant-Intervention-Comparison-Outcomes) format, based on the Joanna Briggs Institute (JBI) (2014) [14], was used to create the criteria inclusion for reviewing the articles. Utilizing any treatment strategies (e.g., usual care, didactic method, participatory learning, internet-based methods) were included in this review. Description of what an inappropriate subject (e.g., articles about diabetes medication alone or intervention that did not include a family component) should include a representative list of reason articles were excluded based on this review. Types of design studies such as single design, descriptive design, qualitative research, no control group, not published in an academic journal (e.g., unpublished dissertation) and studies focused on diabetes prevention or targeting gestational diabetes population were also excluded.

The primary outcome measure was glycaemic control in the past 3 months indicated by patient HbA1c levels. Secondary outcome measures included self-reported on self-care behaviors (e.g., diet, physical activity, blood glucose monitoring, foot care and inspection, and medication adherence), physiological outcomes (e.g., HbA1c, blood glucose level BGL, BP, BMI, lipid profile), and self- reported on levels of self-efficacy and social support from family.

### 3.2. Search Strategy

The search strategy used to find the relevant articles included “type 2 diabetes (T2D),” “self-management,” “diabetes self-management education,” “family support,” “social support” and “uncontrolled glycemic.” Available titles and abstracts of articles were systematically reviewed for their relevance to the topic of DSME involving family support.

### 3.3. Study Selection

This study searched Pubmed, CINAHL, and Scopus databases for articles published between 2008 and 2016. Relevance abstracts were reviewed and duplicate articles were removed.

### 3.4. Synthesis of Results

**Figure 1:**
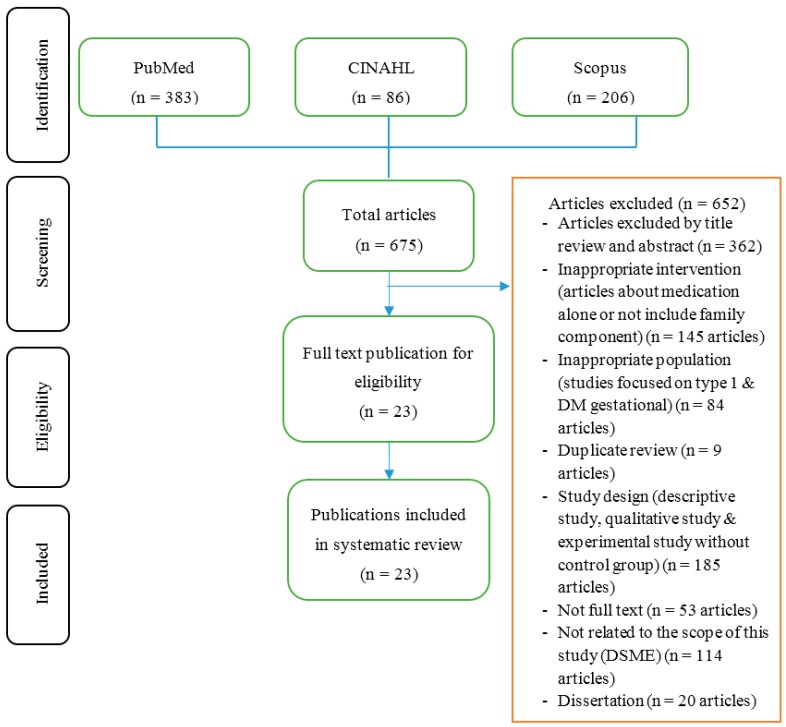
Summary of evidence search and selection.

The results of this review are explained narratively. The description of the results explain: (1) diabetes self-management education (DSME) programs; (2) how to integrate family support in DSME programs; and (3) assess the impact of these programs on health behaviors, physiological outcomes, and clinical health outcomes (Figure 1).

## 4. Results

Twenty-three studies examine the impact of DSME with a family support program on outcomes among uncontrolled glycaemia T2D. Nineteen studies were randomized controlled trials (RCT) [15,16,17,18,19,20,21,22,23,24,25,26,27,28,29,30,31,32,33,34,35], three studies were conducted in the quasi-experimental design [16,36,37], and two studies used a mixed-method design, of which 23 studies were conducted in Western countries [15,16,17,19,20,21,22,25,26,27,28,29,30,32,33,34,35,36,37], whereas only one study was undertaken in Asian countries [24].

### 4.1. DM Self-Management Education (DSME)

We found that 40.9% of the studies [15,16,19,23,26,28,32,33,35] used an individual format and 59.1% used a combination of individuals and group format [15,17,18,20,23,24,30,34,35,37]. In general, both types of program included personalized counseling, goal-setting, problem solving, and explanation of ways in which family members can support self-care practice and follow-up sessions.

#### 4.1.1. Teaching Strategy

In general, DSME was either primarily didactic, such as an educational class offering learning material followed by discussion or they involved more participatory learning (encouraging patients’ active involvement in the learning process, with group discussion sessions, goal setting, negotiation, and problem solving), or they adopted a mixed approach combining both didactic and participatory learning.

16 of the 23 studies combined both didactic and participatory learning with strategies including goal setting, action planning, problem solving and follow-up strategies [17,18,19,21,22,24,25,26,27,30,32,34,35,36,37]. To improve the T2D self-management knowledge and communication skills, one study offered a DVD-based training program [20]. Another study incorporated Bluetooth technology to deliver health information via a supplied modem to the remote secure server. Patients were also given advice on lifestyle modification and how to contact their medical providers [15]. Aikens’ study expanded a DVD-based training program to enhance communication skills [38]. Another study presented a cooking class to demonstrate healthful foods [23]. One study offered diabetes Tele-monitoring calls using the mHealth + CarePartner (CP) to improve diabetes self-management and support blood glucose monitoring [29].

#### 4.1.2. Educational Materials

The education materials in this review varied across different studies. One study used Bluetooth technology to deliver the diabetes information [15]. Four studies used food models, food bingo and food pictures to illustrate healthy diet and food portion size [17,21,23,27]. Diabetes handouts/booklets (diet, exercise, medication, eye and foot self-care) were used to disseminate knowledge [18,19,22,24,33,35,39]. In addition, four studies [16,17,23,37] used video and DVD to demonstrate successful self-care behaviors and practices among patients with diabetes and two studies provided the simple padometer for blood glucose self-monitoring at home [25,28].

#### 4.1.3. Follow-Up

Follow-up strategies are essential components in diabetes care to sustain self-management behaviors. This review verified that 10 studies followed up on their programs in a face-to-face context [15,18,20,25,26,27,30,33,36,37]. Five studies followed up on their programs by telephone [16,17,28,29,35], seven studies mixed face-to-face follow-up and telephone or computer-based follow-up strategies [16,21,22,23,32,35,40].

### 4.2. Integration of Family Support in the DSME Program

Recently, DSME programs have been incorporated into primary care units and the community. Regardless of the setting, the effectiveness of communication and supporting skills are also important in influencing diabetes management behaviors and promoting effective day to day coping [41]. 13 of 23 studies involved patients and family members as a unit intervention and required family members to attend the education classes or meetings [17,18,22,23,24,25,27,29,30,32,34,35,36,37], and 8 studies asked family members to provide support in relieving stress, denial, and maximizing environmental conditions [15,16,19,20,21,26,33,35].

In the study of Keogh et al., family members were needed to assist and support patients in self-management practices by helping patient with strategic planning, goal setting, and problem-solving. Effective feedback concerning negative perceptions of diabetes was used for exchanging health information, reducing care resistance and building self-efficacy emphasized by family members [22].

In other studies, patients received interactive voice response (IVR) calls to monitor the barriers of self-management, medical check-up seeking and connecting with family members. Patients’ improvement record, emotional support as well as problem-solving were also handled by family members [16,38].

A study was conducted using the social cognitive theory, focusing on how environmental change enhances self-efficacy, diabetes self-management and overcoming barriers. Family members emphasized cultural beliefs and values to support dietary change, physical activity, and blood glucose monitoring [17].

One study suggested augmenting communication skills using a speakerphone among couples to support and solve problems of behavior change This program focused on discussion tasks, goal setting and problem-solving to improve the communication skill, achieve goals tailor interventions among uncontrolled glycemia DM2 [42].

In Ananda’s study participants received a mHealth + CarePartner (CP) program to monitor self-management behavior while family members were instructed to provide guidance and support for self-management and help assess barriers to effective self-management behavior [29]. During the program, the summary of result achievements was sent to the patient and their family members to facilitate supportive relationships in order to reinforce the importance of achieving good glucose control.

Regarding emotional supportive behaviors, 7 studies addressed psycho social problems such as feelings of denial, the presence of discordant conditions (e.g., negative response to diabetes self-management, seasonality of mood disorder and the presence of depression in medical illness) and retirement that might occur among patients with diabetes [17,22,25,26,38].

The impact of family support integrated with DSME has been shown to be cost-effective and reduced risks of DM2 complications [13], improved HbA1c levels by as much as 1% [15,18,21,22,24,25,27,35,36] and has a positive effect on psychosocial [38], self-efficacy [17,27], dietary and exercise behavior aspects [22], perceived support [18], knowledge [35], medication adherence and quality of life [35]. It clearly confirmed that DSME with family support is an important factor in controlling blood glucose DM2.

A study comparing diabetes management both with and without family involvement found that patients enrolling with an informal caregiver had a higher rate of engagement and more likely to decrease blood glucose level and to regularly check the blood glucose as well [38]. The practical and emotional support received by family members had a positive influence on global measures of disease management in diabetic patients [10].

Another study reported that elderly patients whose spouses involved with them found greater improvements in knowledge, metabolic control, and stress level compared those who participated alone. Involving family members in the program for elderly patients is important to provide instrumental and emotional support related to diabetes management [13]. The DSME with family support program helped the diabetic patients who had difficulty adhering to diabetes management including educating patients about how to manage living with diabetes; to allow patients to discuss the feeling and accompanying behavior changes associated with diabetes; to facilitate self-esteem and help patients overcome barriers; to develop solutions and techniques to maintain a proper diet; and to help patients develop supportive relationships among family members to maintain diabetes management over time [10,41].

### 4.3. Effectiveness of Family Support Integration on Diabetes Outcomes

DSME with family support has a significant effect on health outcomes according to existing studies in this review. The outcomes have been classified into five categories, including self-management behaviors (diet, physical activity, blood glucose monitoring, foot infection and medication adherence), physiological outcomes, self-efficacy and social support and clinical outcomes (HbA1c/blood glucose level, blood pressure, BMI status, and lipid profile), as described below.

#### 4.3.1. Self-Management Behavior Outcomes

23 articles examined the impact of DSME with family support on self-care behaviors. Strong evidence was given that following good self-management behaviors, including diet, physical activity, blood glucose monitoring, foot inspections and medication adherence significantly improved the clinical outcomes and could prevent long-term complications [5]. 2 studies reported a positive impact on healthy dietary intake [30,34] after receiving the program. One other study showed appropriateness in selecting healthful food and food exchange [20], while 5 studies improved the level of exercise behavior [16,24,30,34,37]. All of those studies showed a high level of family support integration with DSME program.

In addition, when the correlation between perceived support with blood glucose monitoring was examined, 4 studies confirmed that a higher level of support significantly influence blood glucose monitoring at home [16,17,20,23,27,37]. Regarding medication taking behavior, seven studies demonstrated that regular medication adherence was performed under family support [17,18,21,22,23,24]. Furthermore, this review confirmed that increased self-awareness on foot inspections correlated to foot ulcer prevention [16,37].

In contrast to related studies, 4 studies showed medication adherence did not improve significantly in relation to family support [15,23,25,26]. It could be correlated with low family participation [23], understanding of patient and family member roles and patients’ adoption of medication adherence in the long term period [25]. Those opposing results indicated that study limitation had minimized the results.

#### 4.3.2. Psychological Outcomes

As an approach to explore the psychological problems in managing self-care behaviors, barriers related to the psychosocial dimension were reviewed. Emotional responses were associated with poor diabetes self-management and glycemic control, including feeling discouraged by diabetes treatment, worry about long-term complications and incorrectly defining the concrete goals for diabetes care [7]. Twelve of 23 studies reported that higher levels of family support had a positive impact on reducing depressive symptoms [16,18,25,29] and positive emotional control [19], psychosocial well-being [22,34,37], quality of life [29,35] and diabetes related to distress [20]. As a result, less depression had a positive impact on self-management behavior and certain clinical outcomes.

#### 4.3.3. Self-Efficacy

5 review studies explained that patients who have higher perceived support demonstrate significantly better self-efficacy [15,17,19,23,27]. One study received a one-on-one tailored education session and bi-weekly follow-up to influence self-efficacy when performing self-management [35]. This self-efficacy enhancing program could improve self-confidence on diabetes self-management behaviors and glycaemia control.

#### 4.3.4. Social Support Perceived

Social support is a fundamental approach to sustaining self-management behaviors and overcoming barriers among patients with diabetes. 6 experimental studies described the impact of family support interventions to increase perceived social support on self-management among T2D patients [15,16,18,25,26,39]. Individual who receives support is capable of getting advice when coping with difficulties and improving positive communication for diabetes care. It could be a positive impact on a positive relationship between patients and family members on diabetes self-management behaviors. For this reason, social support from family was effective in improving the diabetes self-management behaviors.

#### 4.3.5. Clinical Outcomes

23 articles examined the effect of DSME with family support on clinical outcomes. Ten studies showed the improvement on HbA1c [15,18,21,23,24,25,27,33,34,35] after succeeding in diabetes self-management. 5 studies focused on dietary modification support to improve blood pressure level [15,23,24,29,32], body mass index [15,36], and lipid profile [24,32,35]. The higher level of support has remained a strong factor for the success of self-care behaviors.

In contrast, 2 studies confirmed that there was no significant change in the level of triglycerides and BMI [28]. This could be linked to the low level of peer support over a long-term period. Other studies also showed no significant of changes in HbA1c levels [32,35,37].

## 5. Discussion

We conducted a systematic review of 23 existing studies related to the impact of DSME involving the family as a fundamental source of social support on self-management among T2D patients between 2008 and 2016. The results confirmed the impact of family integration on several health outcomes of T2D. Various studies employed strong study designs such as randomized controlled trials [15,16,17,18,19,20,21,22,23,24,25,26,27,28,29,30,31,32,33,34,35].

DSME is often generated by individual and group education. This strategy offers a combination of didactic teaching and interactive or participatory learning approaches. A collaborative approach to DSME mixed methods of teaching and family support involvement [15,17,18,20,23,24]. The combination of didactic with other methods such as participatory learning, goal setting, action planning and problem-solving had a positive impact on health outcomes and improved health behaviors [16,20,21,22,23,24,26].

This literature review also found that family involvement using a collaborative approach was widely involved across studies. Many studies incorporated the details of family members in program activities such as providing emotional support regarding problem-solving and helping patients to solve their emotional distress or provide information and roles to facilitate, accommodate, remind, motivate and partner with behavior change and perform tasks. Some studies in this literature review found that family members were included in an intervention program. However, they lacked information about how family members provided support of diabetes self-management behaviors, interacted in the program or what family outcomes should be addressed in the intervention. Only a few studies described the role of family members using a participatory learning approach. To effectively engage family members in the intervention, a clear understanding of the theoretical basis of involving family members is needed to serve the T2D patients when performing self-management behaviors.

The duration of the intervention and follow-up was measured using the length of the intervening period from the pretest until completing the program. In the short term, interventions conducted using several strategies, including weekly telephone follow-ups, face-to-face follow-ups, negotiating, and discussing to design the goals and action plan and modify the goals and action plan were more effective in improving health outcomes [15,16,18,20,21,22,23,26].

The follow-up method is an essential component in diabetes self-management among patients with chronic conditions. Generally, follow-up methods are categorized into four strategies, including computer-based, phone call, short message service (mail) and home visits. Different follow-up methods are used to assess the patient’s experience of the program, identify barriers and problem-solving approach to address barriers, to revise the goals and action plan and reinforce any successful performance dietary and exercise self-management. The combination of telephone and face-to-face follow-up is very effective for monitoring the patients’ achievement and its significantly improves the health outcomes by increasing knowledge and self-efficacy to carry out self-management behaviors [16,21,22,23,32,35].

A study that assessed the outcomes found a significant improvement in clinical outcomes such as HbA1c, blood pressure, lipid profile and BMI status after implementing the program. 3 studies mentioned that not significant change of HbA1c level was observed in the short intervening period [20,23,26]. Other issues involving self-care that is often faced by patients with diabetes are to maintain the improved behaviors after the end of the intervention period. However, engaging family members could help patients strengthen self-management interventions and lengthen the effectiveness gained from the intervention [40,41,42,43].

## 6. Strength & Limitations

In this review, we examined many studies involving family members in diabetes self-management with randomized control trial (RCT) designs and some of the studies conducted using a quasi-experimental design. However, some limitations were still encountered. Heterogeneous methods, strategies, populations, settings, and outcomes made it difficult to compare the effect size of each study. Even though we created this systematic review by hand-tracking, there may have been some studies related to family support for diabetes self-management that remain unidentified and excluded because they did not describe family involvement or did not report specific outcomes of diabetes. This review study was limited by publication bias because positive findings tend to be published more frequently than null findings. In addition, the decision to describe the result in a narrative form rather than as a meta-analysis because we did not have access to primary data and the variability in study design did not allow us to pool data. Therefore, we could not describe the effect size of each study outcome.

## 7. Conclusions

Developing diabetes interventions with family support is an integral part of sustaining self-management behaviors and improving the health outcomes of T2D patients. In conclusion, this systematic review found that DSME with family support improves health outcomes for patients with uncontrolled glycaemia. Further study needs to provide details of DSME in the intervention and compare the health outcomes with and without family involvement in DSME programs (Table 1).

## Figures and Tables

**Table 1:** Family support integrated with diabetes self-management and health outcomes.

References	Design	Component of DSME	Integration of Family Support in DSME	Follow-Up	Education Materials	Outcomes
Wild (2016) [15]	Randomized control trial (RCT)	- Providing Bluetooth technology for transmitting readings for patients and family - Advice on lifestyle modification, on lag time for effects of lifestyle and medication change on glucose and blood pressure - Providing information on when and how to contact family practice team via research nurses. - Support	- Family as an informational support to link with the health care provider	- Face-to-face follow up	- Bluetooth technology	- Significant decrease of HbA1c, systolic blood pressure, diastolic blood pressure - No significant changes in weight, treatment pattern, adherence to medication, or quality of life
Garcia (2015) [28]	Randomized control trial (RCT)	- The participants received the DSME including diabetes overview, eating with diabetes, physical activity, managing emotions etc. - Participants received a glucose meter to test blood glucose 3 times per day for six months - Participants were assisted to access the resources needed - Assistance in setting goals and problem-solving	- Families were encouraged to attend the education session at home - The program consisted of eight one-on-one tailored education sessions on topics such as self-management behaviors - Families were assisted to access the resources needed such as accessible clinics	- Telephone follow-up	- Handout at each session - Glucose meter for self-monitoring	- Decreasing HbA1c and improvement of knowledge, self-efficacy, quality of life and LDL cholesterol - There were no significant changes in systolic blood pressure, triglycerides, or BMI
Aikens (2015) [16]	Randomized control trial (RCT)	- Monitored patients’ barriers to self-management - Provided diabetes self-management by using messages - Helped the medical-seeking - Generated the guidance of self-management - DVD-based training in communicating effectively - Questions and feedback messages - Support	- Family members have roles in medical help seeking, and emotional support when patients faced problems	- Telephone follow-up - Short massage service	- DVD - Mail message	- Significant changes in medication adherence, physical functioning, depressive symptoms, and diabetes-related distress - Significant changes in SMBG performance, checking of feet.
Tang (2015) [35]	Randomized control trial (RCT)	- 3-month diabetes self-management education program consisted of 12 weekly 90-min group sessions, a personalized diabetes complications risk profile, one-on-one support activities, face-to-face meetings, self-management goals, develop an action plan and follow-up - 12 months ongoing diabetes self-management support (DSMS) such as emotional and behavioral support in weekly group sessions, follow-up telephone contact - During follow-up, the researcher addressed self-management challenges, evaluated the action plan, problem-solving and developed the future action plan and set the goals	- Peer leader provided the emotional and behavioral support	- Face-to-face follow-up - Telephone follow-up	Not mentioned	- No improvement in HbA1c at 3 months and 15 months - Peer support had significantly lower LDL, systolic blood pressure, diastolic blood pressure, body mass index compared with the DSME-alone group
Hu (2014) [17]	Quasi-experimental	- Providing information related to diabetes exercises and diet - Helped to create healthy eating, monitoring blood glucose and medication taking - Facilitated problem-solving, action plan and discussion with family members about DSM	- Family member was invited to the program and focused on family centeredness - Roles of family in decision-making, problem-solving and emotional support - Building family support which was focused on cultural values	- Telephone call follow-up	- Picture illustration - Flipchart and games - Video tapes - Visual aids	- Significant changes in blood pressure, diabetes self- efficacy diabetes knowledge, and physical and mental components of health-related quality of life - Significant changes in intake of healthy foods and performance on blood sugar tests and foot inspections and lower BMI - No significant changes in levels of physical activity
John et al., (2014) [29]	Randomized control trial (RCT)	- This program is a mHealth + CarePartner (CP) programs - Participants received the weekly automated diabetes telemonitoring calls to include self-management guidance	- The care partners such as family members received email updates on the patient’s diabetes and guidance on supporting their self-management	- Telephone call Follow-up	- Mobile health (mHealth) telemonitoring	- Improvement of glycemic control and diabetes distress - Significant changes in diabetes self-management behaviors, health-related quality of life, systolic blood pressure, and relationship quality
Hamidreza (2014) [18]	Randomized control trial (RCT)	- Assessing educational needs and dividing the family members into small groups based on those needs - 45–60 min of educational sessions about the importance of medication adherence and family support behavior - Teaching for 30–45 min and 15 min and answering questions and exchanging views between family members - Support	- Involving family in education sessions and the exchange of views between family members - Family has role in home blood glucose testing and health behavior such as medication taking	- Face-to-face follow-up	Not mentioned	- Significantly decreased levels of A1c - Significant increase in perceived support - Significant differences in medication adherence and cognitive status
William et al., (2014) [37]	A quasi-experiment	- Inviting the participants to identify their own needs - Enhanced participants’ engagement by showing the videotaped stories of typical problems - Providing information using culturally appropriate material - Helping participants to set the goals and supporting them for self-care	- Families were invited and encouraged to share learning and enhance their ability to support and to know how to be helpful - Families also have a key person to affirm the successful behaviors	- Face-to-face follow-up	- Videotaped	- After 3 months follow-up there was an improvement on psychological and behavioral outcomes, - Increasing knowledge of self-management and personal care skills (exercise and foot care) - Decreasing A1c levels but not significant
Fall (2013) [19]	Randomized control trial (RCT)	- Reflection of personal threat to diabetes care - Providing mastery perception of diabetes - Brief consultation related to positive-emotional group & negative-emotional group	Not mentioned	Not mentioned	Not mentioned	- Significant changes in adherence, perception of diabetes, positive emotion control and greater treatment acceptance
Robling et al., (2012) [20]	Cluster randomized controlled trial	- Promotes shared agenda setting and guiding communication style - Discrete strategies and skill drawn on interview practice - Role played interactions modeled on routine consultations - Education by using didactic and interactive strategies - Practice intervention strategies and skills - Consultations online and to receive feedback	- Family as a facilitator on goal setting, communication, and health-seeking	- Face-to-face follow-up	- Physical tools (3T: TimeToTalk)	- No significant changes to HbA1c - More capable of training staff - No significant skill of practitioner - Reduced problem with treatment barriers - Improve treatment adherence - Greater excitement about clinic visits - Improve continuity of care
Sinclair (2013) [21]	Randomized control trial (RCT)	- Providing education related to diabetes complications - Encouraged to practice self-management - Building on culturally relevant knowledge and skill related to self-care behaviors - Providing communication skills - Action plan on controlling on controlling blood glucose and A1c - Discussion of the challenges of diabetes self-management - Group training by peer educator and facilitator - Storytelling of effectiveness self-management behaviors - Providing support	- Involving family to provide the emotional support and goal setting, problem-solving and health-seeking	- Telephone call follow-up - Face-to-face follow-up	- Images of local foods and physical activity - Individual metaphors - Electronic supplementary materials - Handbook	- Significant changes in A1c levels - Significant changes in knowledge - Significant changes in self-management behaviors
Toobert (2010) [30] Tobert (2011a) [31]	Randomized control trial (RCT)Randomized control trial (RCT)	- The Viva Bien program including weekly meeting and encouraged participants, practice stress management technique, engage in 30 min of daily physical activity, stop smoking, and problem solving-based support group - Cultural adaptation program including the adaptation gathering and focus group, preliminary adaptation design focusing on modified the program, preliminary adaptation test	- Families were involved to family night where family can join in the social support group portion of the meeting, hear a Viva Bien activities - Families also could join to celebrate the participants’ achievement, asked question and answer of those activities	- Face-to-face follow-up	Not mentioned	- The program significantly improved psychosocial and behavioral outcomes (fat intake, stress management, physical activity, social environmental support) at six months - Decreasing the A1c level and health diseases risk factors
Rosal (2011) [27]	Randomized control trial (RCT)	- Addressing of literacy needs and modeling and experiential teaching methods - Cultural tailoring by using an educational soap opera to introduce self-management information and model attitudinal change - Reinforce information taught, emphasis on making traditional food healthier - Increasing the walking steps by using a step counter - Brief personalized counseling, goal setting and problem-solving	- Families were invited to attend the home-based support group meals - Family were also invited to discuss the way how to implement the recipes at home and acceptability of family at home	- Face-to-face follow-ups	- Food bingo, and Soap opera - A color-coded graph - Glucose meter and simple logs for tracking the glucose value	- Significant changes in HbA1c at 4 months follow-up but no change at 12 months follow-ups - Significant of self-efficacy, blood glucose monitoring, and diet
Keogh (2011) [22]	Randomized control trial (RCT)	- 16 h of training in motivational interviewing - 15-min follow-up telephone call - Individually tailored to participants’ needs and attempted (clarify negative perception, personalized action plan, mobilized family support) - Building self-efficacy, problem-solving, and goal setting/action	- Involving family member on emotional support and problem solving	- Telephone follow-up call - Face-to-face follow-up	Not mentioned	- Significant changes to A1C levels - Improved beliefs about diabetes, psychological well-being, diet, exercise, and family support.
Kang (2010) [23]	Randomized control trial (RCT)	- Three brief individual education sessions at 1, 3 and 5 months related to diabetes, eating habits, physical activity, and foot care - 2-day long group education session at 2 and 4 months related to demonstrating of cooking, exercise, and foot care - Diabetes sharing groups - A monthly 25–30 min telephone discussion	- Emphasizing family participation on goal setting, gaining the knowledge and improve the skill	- Telephone follow-up - Face-to-face follow-up	- Diabetes hangout - Food models - Cooking books - Exercise video and DVD - Oral medication pictures & power point - Foot massage pictures	- Significant changes to family supportive behaviors - Significant changes to patients’ knowledge and attitude - Not significant A1c, lipid profile - Not significant changes to self-care behaviors
Tai (2010) [36]	Mix methods	- Meeting of the family education diabetes series program. The meeting is begun by checking the blood sugar, weight and foot inspections - Discussion on meal’s ingredients, portion size, and healthy weight maintained - Planning and design a priority of activities based on participants’ interest - Informal sharing information and support	- Family members were invited to attend the family education diabetes series program, - Involved in the data collection but not enrolled as participants	- Face-to-face follow-ups	Not mentioned	- The data show significant in weight, blood pressure, and HbA1c level.
Kluding (2010) [24]	Pretest-posttest single group	- 3 to 4 days per week exercise sessions included stretching exercise to warm up and cool down with a 20-second hold and deep breathing - Weekly nutrition session included decreasing fat intake, increasing consumption of fruits, vegetables, and whole grain - Education session related to setting goals, monitoring ABCs of diabetes, healthy feet, family support day, stress management, preventing depression and building healthy relationship - Family support	- Family member and other supportive were included in the education session especially in family support day and graduation ceremony	Not mentioned	- Food labels - Informational material - Cardiovascular training equipment	- Significant changes to A1c and pain - Significant changes to self-efficacy
García-Huidobro (2011) [25]	Randomized control trial (RCT)	- Interdisciplinary family meetings or home visits - Received a recipe book for diabetes and during family meetings, they received a framed family picture. - Individual counseling session and one counseling session (Learn about strategy of caring) - Multifamily group sessions (trained in motivational interviewing and family counseling and clinicians that guided multifamily groups)	- Emphasizing family involvement on family meeting to discuss the psychosocial problem and health behavior strategies	- Face-to-face follow-up	- Recipe book - Framed family picture	- Significant changes to HbA1c and reduce of depressive symptoms - No significant changes in family functioning style, health behaviors, medication adherence and knowledge of diabetes
Gary (2009) [32]	Randomized control trial (RCT)	- The intervention consisted of two intervention groups such as minimal intervention, a telephone-based intervention and an intensive intervention consisting of education and follow-up service - Minimal intervention included the 6 months remaining of preventive health screening (HbA1c, primary care, and specialty visit) - Intensive intervention was conducted 6 weeks such as guidelines and practical information, self-management education, home-based assessment and education, field experience, skill reinforcement and maintenance and quality control	- Families were involved to provide additional diabetes education - The CHW monitored participant and family behaviors, reinforcing adherence to diabetes treatment	- Telephone call follow-up for - mailing based follow-up - Face-to-face follow-up at clinic ant at home	Not mentioned	- Increasing HDL level and decreasing diastolic blood pressure - No significant HbA1c changes
Vincent (2008) [26]	Randomized control trial (RCT)	- 2-h group sessions, didactic: cooking demonstrations, and group support sessions. - Culturally tailored: low- fat modifications, discussing of home remedies, and appropriate exercise strategies: walking and dance - To bring a family member as a support person	- Family as support system on emotional support and performing self-management behaviors	- Face-to-face follow-up	Not mentioned	- Significant changes to weight and BMI - Improve frequency of self-glucose monitoring and physical activity - No significant changes to diet, foot care, or medications, knowledge, self-efficacy, blood glucose, and HbA1C
Utz (2008) [33]	Randomized control trial (RCT)	- Culturally relevant group DSME intervention consisted of eight 2-h education session over an 8 week period, incorporated the activities and problem solving in each group session - DSME was delivered by emphasizing the supportive atmosphere, storytelling related to diabetes care, diabetes education material, lesson, an approach emphasizing respect and empowerment - Individual DSME session: week 1 is setting goals; week 4 is reviewing the progress, problem solving, offering additional information; and week 8; final review, discussion about the self-management achievement and plan for future self-care	- Families were invited to selected group session to gain the diabetes information and peer support - Families had an opportunity to watch the video regarding to family communication on diabetes care - Discussion and sharing experiences related to diabetes care - Families also were invited in cooking demonstrating activities and how to cook healthy meals	- Face-to-face follow-ups	- Colorful 1-page Handouts - Brochure - Cook book, exercise videotape, pedometer and foot care kit	- DSME improved the self-care activities, A1c level, and goal attainment
Islam et al., (2012) [41]	Mix method	- Focus group moderation among and survey administration conducted 6 focus groups with 47 Bangladeshi women and men living to gain an in-depth understanding of health beliefs, behaviors, and barriers to and facilitators of diabetes management. - CHWs also encouraged family support during the one-on-one visits	- Focus group findings led to the inclusion of family and addressing family support in the intervention. - This focus group was used to overcome family conflict and promote positive family communication and family activities to promote social support would be incorporated into the intervention	- Face-to-face follow-ups	Not mentioned	- HWs qualitatively reported having an impact on family members and on facilitating family support between participants and their family members regarding positive communication and relationship - 87% of the diabetic focus group participants and 95% of the diabetic community respondents did not know the meaning of hemoglobin (Hb) A1c (an important indicator of diabetes control). - 23% of the focus group participants and 12% of the community respondents reported uncontrolled HbA1c levels, but the majority in both groups were not able to report their HbA1c
